# Genotoxicity and cytotoxicity effects of x-rays on the oral mucosa epithelium at different fields of view: A cone beam computed tomography technique

**DOI:** 10.22088/cjim.14.1.121

**Published:** 2023

**Authors:** Roghaieh Faeli Ghadikolaei, Hakimeh Ghorbani, Maryam Seyedmajidi, Kourosh Ebrahimnejad Gorji, Ehsan Moudi, Seyedali Seyedmajidi

**Affiliations:** 1 Student Research Committee, Babol University of Medical Sciences, Babol, Iran; 2 Oral Health Research Center, Health Research Institute, Babol University of Medical Sciences, Babol, Iran; 3 Dental Materials Research Center, Health Research Institute, Babol University of Medical Sciences, Babol, Iran; 4 Department of Medical Physics Radiobiology and Radiation Protection, School of Medicine, Babol University of Medical Sciences, Babol, Iran

**Keywords:** Cone beam computed tomography (CBCT), Oral mucosa epithelium, Genotoxicity, Cytotoxicity, Field of view (FOV)

## Abstract

**Background:**

Cone beam computed tomography (CBCT) is considered a common examination for dentistry problems. Cellular biology can be affected by exposure to ionizing radiations procedures. In this study, we aimed to assess the genotoxicity and cytotoxicity effects of CBCT dental examinations at two different fields of view (FOVs) in exfoliated buccal epithelial cells.

**Methods:**

Sixty healthy adults participated in the current study. They were divided into two identical groups; CBCT with FOV of 6*6 cm2 and 8*11 cm2. Exfoliated oral mucosa cells were prepared immediately before and after 10-12 days of CBCT exposure. The cytological smears were stained with the Papanicolaou technique. The amounts of micronuclei and other cytotoxicity cellular changes (Pyknosis, Karyolysis, and Karyorrhexis) were evaluated. The variables of the parameters before and after CBCT examination in the two investigated FOVs were performed using Wilcoxon test and paired-samples t-test in SPSS software.

**Results:**

The micronuclei and other cytotoxic changes parameters before and after CBCT exposure for both FOVs (6*6 and 8*11 cm2) increased significantly (p<0.001). Furthermore, a significant difference (p<0.05) was observed between the investigated parameters at the two FOVs. Notably, the FOV of 8*11 cm2 had more side effects than that of 6*6 cm2. There were no statistically significant among males and females for both FOVs.

**Conclusion:**

CBCT examinations of dental disorders would increase the risks of inducing genetic damage. The cytotoxicity and chromosomal damage were considered in males and females in both investigated FOVs (6*6 and 8*11 cm2). In this regard, the use of CBCT must be following the ALARA principle.

Cone beam computed tomography (CBCT), due to the high quality and diagnostic accuracy, helps dental practitioners diagnose (1,2). Three-dimensional (3D) images of dental structures along with clear images with highly contrasted forms are the main advantages of CBCT method (3). Compared with multidetector computed scan (MDCT), CBCT technology offers several advantages in clinical practice, such as image accuracy, low dose of the radiation, rapid scan time, and real-time analysis (1,4). In addition, CBCT can show the depth of the structure, mostly of the mineralized tissues, like bone (5,6). It also overcomes the conventional radiographs due to the obtaining 3D information of the images (5). However, there are significant concerns about the side effects of ionization radiation related to the use of CBCT. The organ dose, which is the absorbed dose, is exposed especially the salivary glands, the head and the neck organs (7).

The field of view (FOV) size is one of the main important factors which affects patient dose in CBCT (8,9). Hence, choosing the appropriate FOV size matching the interest area provides a considerable dose saving. Additionally, image quality, contrast, and artifact production could be affected by FOV due to x-ray scatter. All in all, FOV sizes can be various by several centimeters in height and cross-sectional diameter, and their choice involves the necessary planning and diagnosis (10). In a way, a smaller FOV is adequate; however, large FOVs are utilized commonly for orthodontic treatment and/or orthognathic surgery (11). Therefore, different FOVs offered by the manufacturer for CBCT machines should be assessed. It was reported that even a low dose of ionization radiation may cause to cytotoxic effects and DNA damage, therefore, the use of CBCT must be considered by clinical justification (11). X-rays are clastogenic agents and induce the formation of micronuclei, as well as cytotoxic changes which account as sensitive biomarkers to evaluate the side effects of ionization radiations. These genetic effects may include changes in the value and structure of chromosomes and mutations firmly related to cancer development (5,12). The micronuclei are a reliable test for evaluating mutagenicity in human tissues. This test is based on the formation of micronucleus from particles of chromatin material, so the damaged or dysfunctional spindle chromosomes cannot migrate towards the poles during anaphase and are not incorporated into the nucleus of the telophase of a dividing cell (13). The cytotoxicity, which refers to toxic factors and usually causes lethal damage or necrosis, leads to nuclear changes such as Pyknosis (chromatin condensation), Karyorrhexis (fragmentation of Pyknotic nuclei), and Karyolysis (dissolution of chromatin). For the micronucleus test process, peripheral lymphocytes are used commonly; however, the use of exfoliated buccal epithelial cells is of particular interest due to its low cost and non-invasive, simple to perform and analyze, and has a high correlation with the lymphocyte micronucleus assay (14, 15). Several relevant studies investigated CBCT side effects based on exfoliated buccal cells. For example, Mounika et al. (16) assessed the genomic damage from buccal epithelial cells in patients exposed to CBCT. In another study (17), the authors assessed DNA double strand breaks in buccal mucosal after CBCT examination. In both investigations, the side effects of CBCT examination have been approved.

In the current study, we assessed the frequencies of micronucleus cells and monitored cytotoxic effects, Pyknosis, Karyorrhexis, and Karyolysis, in exfoliated buccal mucosal cells of adults following different FOVs of CBCT exposure.

## Methods

**Participants: **In this study, 60 healthy adults exposed to CBCT examinations for dental disorders were included. Informed consent has been received from the participants, and the project was approved by a National Ethics Committee with the registration number of IR/MUBABOL/REC/1399/471.

The exclusion criteria were (a) systemic diseases such as leukemia, lymphoma, rheumatismal diseases, and diabetes mellitus; (b) history of treatment like radiation therapy of head and neck area and immunosuppressive or cytotoxic drugs; (c) localized pathologic changes such as macroscopic abnormalities, gingival and periodontal diseases; (d) individuals with harmful habits like smoking and alcohol consumption; (e) individuals who were affected to infectious or inflammatory diseases; (f) pregnant women; and (g) persons with removable dentures.

**CBCT** **examination:** All CBCT examinations were carried out at one public establishment of dental radiology. Individual characteristics such as gender and age were collected. A dentist requested all CBCTs and performed using X MIND (ACTEON Olgiate olona Italy) device. The scanning procedures were applied in the two different protocols, depending on the patient’s genders. The parameters related to the tube voltage and tube current were 90 kVp and 8 mA for men, respectively, as well as 85 kVp, and 8 mA for women. Notably, the site of choice was the posterior region mandible. The participants were divided into two identical groups based on the different fields of view (FOVs); the FOV of 6*6 cm^2^ and 8*11 cm^2^. 

**Collection of the exfoliated oral mucosa: **Oral mucosal epithelial cells were collected directly before and after 10-12 days of CBCT exposure. The participants were asked to wash their mouths with water prior the preparation of the cytologic smears. Then, the smears were prepared with exfoliating cytology method utilizing cytobrush (Papette®, Wallach Surgical Devices, USA). The cytobrush was placed in contact with the area of the oral epithelium with a constant mean pressure of 10-17 times. Notably, the pressure of the hand during the preparation of the smear was such that only the superficial epithelial cells of the mouth were isolated, and no bleeding occurs. The cytological smears were stained with the Papanicolaou technique within a maximum of three days (18). Ten steps were performed to stain the cytological samples: 1) placing in graded alcohol series (90˚, 70˚, and 50˚), 2) placing in distilled water, 3) staining with hematoxylin for 5-10 minutes, 4) placing in distilled water followed by acid alcohol (0.5%), 5) exposing to distilled water and lithium carbonate, 6) rinsing with distilled water, 7) placing in graded alcohol series (50˚, 70˚, and 90˚), 8) putting in an orange solution for one minute, alcohol (95˚) and absolute alcohol, 9) fixing in xylene, and 10) mounting on glass and cover with glass stopper (19).

**Genotoxicity** **and cytotoxicity** **assessment: **For qualitative evaluation of genotoxicity and cytotoxicity effects, specimens were assessed under light microscope (Olympus BX41, Olympus Corporation, Tokyo, Japan) with 400 x magnification. A total of 100 cells were scored per patient for each sampling time (before and after x-ray exposure) against a background of no bleeding, necrosis, or exudate (9). The selected cells should have proper staining and defined cytoplasmic borders; if we observed overlapping of cells and indistinct cellular membranes, those cells were not included in the study. An experienced and blinded cytopathologist analyzed all the slides.

**Criteria for identifying micronucleus: **The micronuclei were scored based on the Sarto et al. described criteria (20) as a parameter for DNA damaging (mutagenicity). The criteria for abnormality of micronucleus formation were included; less one-third of the diameter of the primary nucleus, not optically refractive (to exclude small dye particles), color similar to or lighter than the nucleus (to exclude large staining particles), located further away from the smaller nucleus 3 or 4 nucleus diameters and without touching the nucleus (to make meaningful frequency measurements), and not more than 2 micronuclei associated with a nucleus (21). The following nuclear alterations were considered for cytotoxicity effects: Pyknosis, Karyolysis, and Karyorrhexis (22,23).

**Statistical** **analysis: **Wilcoxon test and paired-sample t-test were used to compare micronucleus frequency and other cytotoxic changes among the samples before and after CBCT examinations. This process was performed in SPSS software (Version 18, IBM, USA). Statistical significance level was set at 5%.

## Results

The 60 investigated healthy adults were 16 males (35.87±9.56 years) and 14 females (37.14±9.41 years) that referred to the radiology department for CBCT examination with the FOV of 6*6 cm^2^. In addition, for the FOV of 8*11 cm^2^, 13 males (33.23±7.54 years) and 17 females (35.24±7.97 years) were referred.

**Figure 1:**
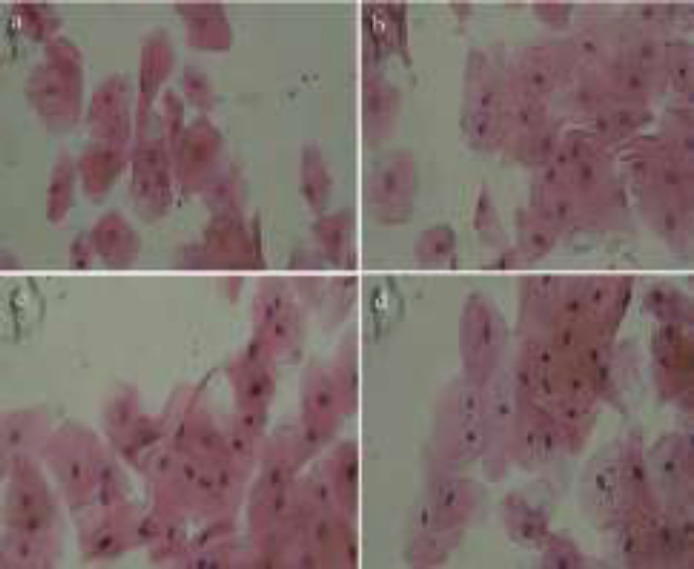
Sample of microscopic imaging of smears stained by Papanicolaou; before (a) and after (b) CBCT scans with the FOV of 6*6 cm2, and before (c) and after (d) CBCT scans with the FOV of 8*11 cm2

**Figure 2:**
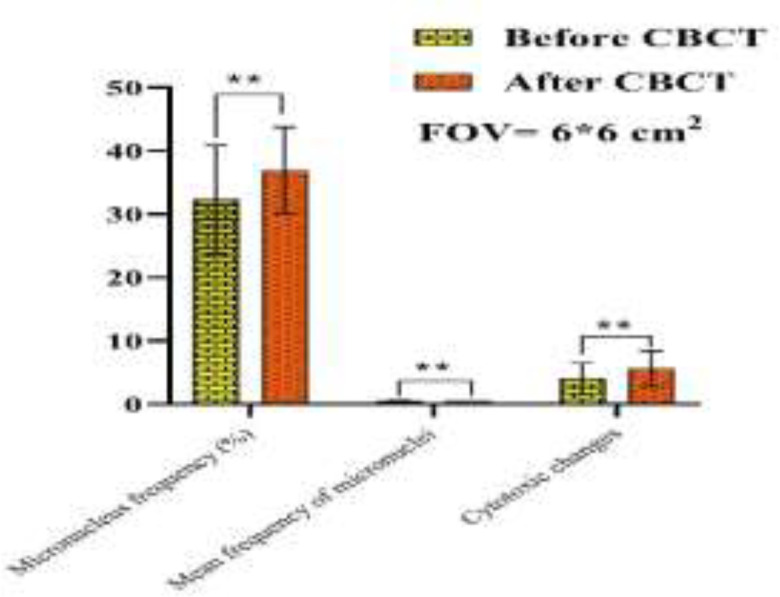
The differences of micronuclei and other cytotoxic parameters before and after exposure of CBCT at FOV of 6*6 cm2. ** Sign defines as P-value<0.001

**Figure 3.**
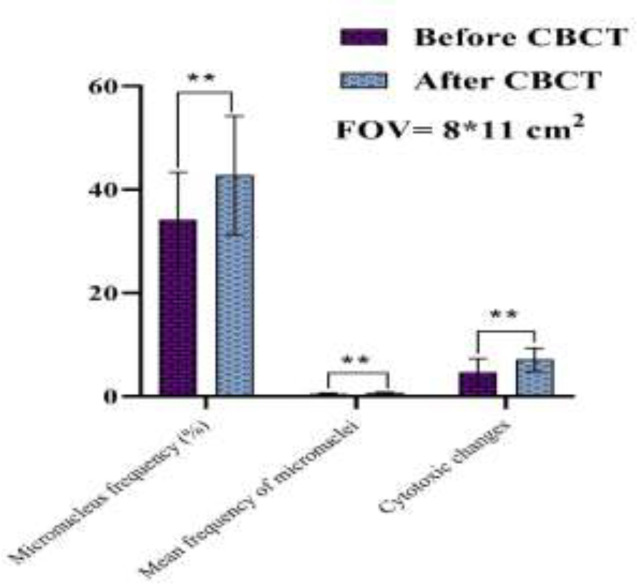
The differences of micronuclei and other cytotoxic parameters before and after exposure of CBCT at FOV of 8*11 cm2. ** Sign defines as p<0.001

**Figure 4:**
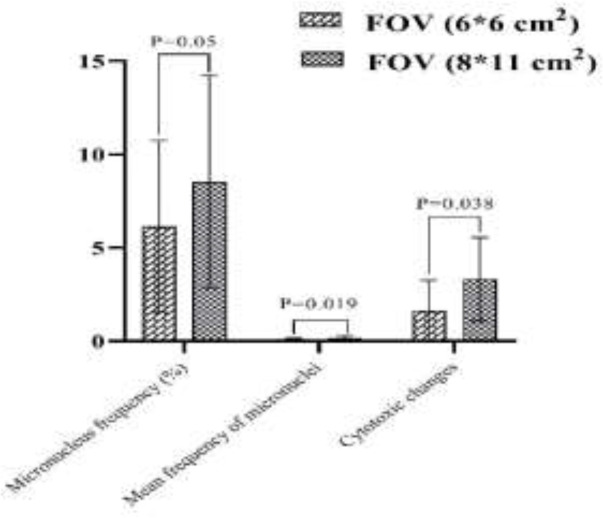
The differences between the FOVs of 6*6 cm2 and 8*11 cm2 for micronuclei and other cytotoxic changes after CBCT examinations

The samples of microscopic imaging of smears stained by Papanicolaou before and after examinations of CBCT in the two different FOVs (6*6 and 8*11 cm^2^) are shown in figure 1. As depicted in figures 2 and 3, the micronuclei and cytotoxic change (Pyknosis, Karyolysis, and Kariorrhexis) values after exposure of CBCT for both FOVs increased significantly (p<0.001). In addition, the differences of the two investigated FOVs based on the mean percentage variations in micronucleus frequency, mean value of micronuclei in each cell, and cytotoxic changes after CBCT exposure were significant (P<0.05). In a way, the FOV of 8*11 cm^2 ^had more side effects than that of 6*6 cm^2^ (figure 4).

As depicted in table 1, based on the micronuclei and other cytotoxic parameters, there was no significant difference (p>0.05) in the outcomes of men and women for the two FOVs studied.

**Table 1:** The differences related to micronuclei and other cytotoxic changes after exposure of CBCT examinations in the two FOVs among the two genders

**FOV of CBCT examination**	6*6 cm ^2^			8*11 cm ^2^		
	**Male**	**Female**	**P-value**	**Male**	**Female**	**P-value**
Micronucleus frequency (%)	9.6±5.9	11.2±4.6	0.02	15.6±9.1	17.52±11.45	0.2
Mean frequency of micronuclei	1.3±1.8	1.85±1.35	0	2.61±2.53	3.82±2.21	0
Cytotoxic changes	5.5±3.1	8.4±3.5	0.02	7.1±5.41	9.58±5.81	0.03

## Discussion

The frequencies of micronucleus cells and cytotoxic alternations including Pyknosis, Karyorrhexis, and Karyolysis were assessed in exfoliated buccal mucosal cells before and after using different FOVs of CBCT dental examinations. The sample size was selected based on previous studies (24–28), in a way that, most of the investigations, the samples were chosen 30; however, we tried to choose more patients (60 samples) to detect small changes with more statistical confidence. Several advantages are reported for using exfoliated buccal cells instead of peripheral lymphocyte for micronucleus assessment, such as, buccal cells under direct radiation exposure at CBCT examination, ease of analysis, reasonable cost, non-invasive, speed performance, and high accuracy (1,14). Notably, the collections of the basal buccal cells were performed after 10-12 days (29) of CBCT examinations in the current study, because following the previous investigations, the period of coming up to the surface and exfoliation of these epithelial cells was about 7-16 days (30, 31).

Our finding demonstrated a significant increase of micronuclei cells and other nuclear alterations (the average value of Pyknosis, Karyorrhexis, and Karyolysis) after 10-12 days of CBCT scans for both investigated FOVs (6*6 and 8*11 cm^2^). In line with the current work, several studies stated that CBCT could increase genotoxicity and cytotoxicity in oral cells of adults, generally (2, 12, 30, 32, 33). For instance, Basha and Essawy, 2018 (33) demonstrated that the frequency of micronuclei increased significantly after CBCT exposure (0.026 to 0.030, p <0.001). Also, the other nuclear alterations showed a significant increase (0.013 to 0.027, p<0.001). In addition, da Fonte et al., 2018 (5) expressed that a significant difference was found in the number of micronucleated cells and cytotoxicity (p< 0.001). Different results related to the number of micronuclei cells and other nuclear changes may be attributed to different use of radiation parameters (kV and mA), population features, and various types of devices and methods. Additionally, other factors such as age, lifestyle, oral hygiene (e.g., use of mouthwash), smoking, and alcohol can effect on this cell monitoring (34). In a way, the studies have shown that smoking, regular alcohol consumption, and continued use of mouthwash increase the frequency of micronucleus in oral cells (34–36). 

Notably, these factors are considered as the exclusion criteria in our study. Moreover, since each patient was considered their own control, any potential difference between the first and second counts could be due to radiation. As expected, the larger FOV (8*11 cm^2^) would induce higher variations because of the higher patient dose received. In a study by Nascimento et al., 2017 (37), they have compared the absorbed dose among the three various CBCT FOVs (5*5, 6*8, and 8*15 cm^2^) for evaluation of temporomandibular joints. They stated that FOV had a large effect on absorbed dose, in a way, dose reduction occurred when using small FOV, but there was no linear relationship between FOV size and dose. In addition, there are other factors like kVp, mA, and voxel size (33), which affect the dose received; as a result, we standardized the scanning protocol for all patients. All in all, for large FOV, thyroid shielding is recommended for patients to aid further dose reduction, and genotoxicity and cytotoxicity side effects. In addition, CBCT FOVs that are smaller and reproduce high definition at a very low dose while maintaining sufficient image quality are generally recommended. In the current work, we have selected the identical number of participants for both FOVs of CBCT examination, and also the number related to the gender was similar approximately. Additionally, the patients were chosen in one province to minimize any epidemiological differences in radiation sensitivity. No significant difference was observed among the genders for micronucleus scores and other cellular changes in both investigated FOVs. Popova et al., 2007 (29) investigated the genotoxic effect of panoramic radiography in buccal epithelium cells. Although there was no significant increase in the frequency of cells with micronucleus test after panoramic tomography, a significant correlation was observed between the ages of the subjects studied. The current study’s limitations are listed which can be performed in future study. The toxic effects of CBCT on the buccal mucosa could be compared with the children group and other dental radiography modalities like MDCT. In addition, larger sample size could be considered at various radiobiological tests like comet tail assay or Gamma H2ax. The finding of our study demonstrated that CBCT examinations of dental disorders using both investigated FOVs (6*6 and 8*11 cm^2^) can induce genotoxicity and cytotoxicity side effects in the buccal mucosa which was not gender-related. ALARA (as low as reasonably achievable) principle should be followed for the use of CBCT examinations for the diagnosis of dental anomalies. Notably, thyroid shielding is recommended for patients during CBCT scans, especially in large FOV (e. g., 8*11 cm^2^).
